# Emerging trends in Lassa fever: redefining the role of immunoglobulin M and inflammation in diagnosing acute infection

**DOI:** 10.1186/1743-422X-8-478

**Published:** 2011-10-24

**Authors:** Luis M Branco, Jessica N Grove, Matt L Boisen, Jeffrey G Shaffer, Augustine Goba, Mohammed Fullah, Mambu Momoh, Donald S Grant, Robert F Garry

**Affiliations:** 1 Autoimmune Technologies, LLC, New Orleans, Louisiana, USA; 2 Department of Microbiology and Immunology, Tulane University, New Orleans, Louisiana, USA; 3 Corgenix Medical Corporation, Broomfield, Colorado, USA; 4 Department of Biostatistics and Bioinformatics, Tulane University, New Orleans, Louisiana, USA; 5 Lassa Fever Laboratory - Kenema Government Hospital, Kenema, Sierra Leone; 6 Eastern Polytechnic College, Kenema, Republic of Sierra Leone; 7 Ministry of Health and Sanitation Workplace Health, Freetown, Republic of Sierra Leone; 8 Kenema Government Hospital Lassa Fever Ward, Kenema, Republic of Sierra Leone

## Abstract

**Background:**

Lassa fever (LF) is a devastating hemorrhagic viral disease that is endemic to West Africa and responsible for thousands of human deaths each year. Analysis of humoral immune responses (IgM and IgG) by antibody-capture ELISA (Ab-capture ELISA) and Lassa virus (LASV) viremia by antigen-capture ELISA (Ag-capture ELISA) in suspected patients admitted to the Kenema Government Hospital (KGH) Lassa Fever Ward (LFW) in Sierra Leone over the past five years is reshaping our understanding of acute LF.

**Results:**

Analyses in LF survivors indicated that LASV-specific IgM persists for months to years after initial infection. Furthermore, exposure to LASV appeared to be more prevalent in historically non-endemic areas of West Africa with significant percentages of reportedly healthy donors IgM and IgG positive in LASV-specific Ab-capture ELISA. We found that LF patients who were Ag positive were more likely to die than suspected cases who were only IgM positive. Analysis of metabolic and immunological parameters in Ag positive LF patients revealed a strong correlation between survival and low levels of IL-6, -8, -10, CD40L, BUN, ALP, ALT, and AST. Despite presenting to the hospital with fever and in some instances other symptoms consistent with LF, the profiles of Ag negative IgM positive individuals were similar to those of normal donors and nonfatal (NF) LF cases, suggesting that IgM status cannot necessarily be considered a diagnostic marker of acute LF in suspected cases living in endemic areas of West Africa.

**Conclusion:**

Only LASV viremia assessed by Ag-capture immunoassay, nucleic acid detection or virus isolation should be used to diagnose acute LASV infection in West Africans. LASV-specific IgM serostatus cannot be considered a diagnostic marker of acute LF in suspected cases living in endemic areas of West Africa. By applying these criteria, we identified a dysregulated metabolic and pro-inflammatory response profile conferring a poor prognosis in acute LF. In addition to suggesting that the current diagnostic paradigm for acute LF should be reconsidered, these studies present new opportunities for therapeutic interventions based on potential prognostic markers in LF.

## Background

LASV is a member of the *Arenaviridae *family and is the etiologic agent of LF, which is an acute and often fatal illness endemic in West Africa. There are an estimated 300,000 - 500,000 cases of LF each year [1-7] with a reported mortality rate of 15%-20% for hospitalized patients. Mortality rates for LF can become as high as 50% during epidemics [3,8,9] and 90% in third trimester pregnancies for both the expectant mother and the fetus. Presently, there is no licensed vaccine or immunotherapy available for prevention or treatment of this disease. The severity of LF, its ability to be transmitted via aerosol droplets [10], and the lack of a vaccine or therapeutic drug led to its classification as a National Institutes of Allergy and Infectious Diseases (NIAID) Category A pathogen and biosafety level-4 (BSL-4) agent. The antiviral drug ribavirin has been demonstrated to reduce fatality from 55% to 5%, but only if it is administered within 6 days after the onset of symptoms [1,8,9]. There is currently no commercially available LF diagnostic assay, which presents a major challenge for early detection and rapid implementation of existing treatment regimens.

Since 2005, continuous infrastructure improvements at the KGH Lassa Fever Laboratory (LFL) by the National Institutes of Health (United States), the Department of Defense (DoD), the Naval Facilities Engineering Command (NAVFAC), the United States Army Medical Research Institute of Infectious Diseases (USAMRIID), the World Health Organization (WHO), Global Viral Forecasting (GVF) and Tulane University have resulted in the implementation of sophisticated, on-site diagnostic and research capabilities [11,12]. Currently, LF is diagnosed at the KGH LFL using ELISA and lateral flow immunoassays (LFI) that detect viral Ag. Virus-specific IgM and IgG levels are also determined in serum samples for all suspected cases that present to the KGH LFW. Additionally, the laboratory assesses 14 serum analytes using a Piccolo^® ^blood chemistry analyzer coupled with comprehensive metabolic panel disks. Flow cytometry powered by a 4-color Accuri^® ^C6 cytometer performs immunophenotyping, intracellular cytokine and bead-based secreted cytokine analysis on patient sera. These resources contributed to advances in real time diagnosis along with metabolic and immunological characterization of acute LF, thus resulting in a marked improvement in the management of the disease. Herein we present evidence that introduces new insight into humoral and cellular immune responses to LASV that have lead us to reevaluate the role of LASV IgM seropositivity in diagnosing acute LF in suspected cases living in the LASV endemic areas of West Africa. An improved understanding of the natural history of LF will be helpful in guiding future research in diagnosis and treatment.

## Methods

### Human Subjects

Suspected LF patients, individuals reporting close contact with confirmed LF patients, and healthy volunteers were eligible to participate in these studies as outlined in Tulane University's Institutional Review Board (IRB) protocol, National Institutes of Health/National Institutes of Allergy and Infectious Diseases (NIH/NIAID) guidelines governing the use of human subjects for research, and Department of Health and Human Services (HHS)/NIH/NIAID Challenge and Partnership Grant Numbers AI067188 and AI082119 and HHS Contract HHSN272200900049C. The Tulane University IRB has approved these projects. All subjects participating in the study gave written informed consent to the publication of their case details.

### Sera from suspected LF patients and healthy volunteers

Small blood volumes (typically five milliliters [mL]), for serum separation were collected from study subjects with consent from the attending physician. Blood from healthy Sierra Leonean volunteers were used as normal controls. Three groups of normal donors were assembled for this study: 1) non-febrile volunteers comprised of Lassa program staff from Kenema district and nursing staff from a hospital in Bo, Sierra Leone, who reported to be in generally good health at the time of blood collection; 2) volunteers from randomly-chosen, historically non-endemic villages in the District of Moyamba (N = 101) and the District of Bombali (N = 13) that also reported to be in generally good health, not having any recent illnesses or having travelled to Africa's Eastern Province. Both patients and healthy volunteer samples received a coded designation and were collected in serum vacutainer tubes and allowed to coagulate for 20 minutes at room temperature. Serum was separated from coagulated blood by centrifugation (200 × *g*, 20 minutes at room temperature). The serum fraction was collected for analysis, and aliquots were stored in cryovials at -20°C. Sera from a panel of 50 randomized U.S. male donors were purchased from Bioreclamation, Inc. (Westbury, NY), and a similar panel of 50 U.S. female donors was obtained from SeraCare Life Sciences, Inc. (Milford, MA). All U. S. donors were at least 18 years of age and reported their ethnic groups as Caucasian, Hispanic, or African American.

### Patient database analysis

A database was generated with coded patient designations and corresponding LASV antigen, IgM and IgG status at the time of testing and admission. In total 1,909 suspected LF patient data were collected and a subset of those data were used in this study. All patients in the database were suspected of having LF based on clinical presentation, or for failure to respond to anti-malarial and/or antibiotic drug regimens over the course of a prolonged febrile illness.

### Detection of LASV antigen by LFI, ELISA and PCR

Serum levels of LASV nucleoprotein (NP)-specific Ag were measured using LASV Antigen Rapid Test cassettes and dipstick LFI currently under pre-clinical development by Corgenix Medical Corp. (Broomfield, CO) and the Viral Hemorrhagic Fever Consortium (see acknowledgements), as described by Grove et al. [11]. Positive LF diagnosis was confirmed with a sensitive Ag-capture ELISA employing either a murine monoclonal or caprine polyclonal capture antibody (Autoimmune Technologies, L.L.C., New Orleans, LA) followed by a peroxidase-labeled caprine reagent and tetramethylbenzidine (TMB) substrate, as previously outlined by Grove et al. [11]. Both methods were substantiated with a RT-PCR test. RNA was extracted from serum using a QIAmp Viral RNA Mini kit (QIAGEN, Valencia, CA). RT-PCR was performed using SuperScript III (Invitrogen, Carlsbad, CA) with primers 36E2 and 80F2 or LVS-339-rev directed against the LASV glycoprotein complex (GPC) gene for amplification of a highly conserved 318 nucleotide fragment (Lassa virus Josiah positions 4 to 322 of S RNA) [13].

### Detection of LASV-specific serum IgM and IgG levels by ELISA

Individual recombinant LASV (reLASV) proteins (Vybion, Inc., Ithaca, NY; The Scripps Research Institute, La Jolla, CA; AutoImmune Technologies, LLC, New Orleans, LA; Tulane University Health Sciences Center, New Orleans, LA) and combinations of reLASV proteins optimized for detection of virus-specific IgM and IgG levels in serum were coated in stripwell plates at Corgenix Medical Corp. and packaged as test kits. Detection of LASV-specific IgM and IgG in suspected LF patient and normal control sera was performed as previously outlined by Grove et al. [11]. IgM binding specificity in Moyamba and Bombali normal donors was confirmed by western blot on LASV NP and by competition assays with sera spiked with recombinant NP in ELISA. Similar assays were performed to ascertain IgG binding specificity. Additionally, normal and LF patient sera were depleted of IgG with ProteaPrep IgG Depletion Sample Prep Kits (Protea, Morgantown, WV), as per manufacturer recommendations. Binding of LASV-specific IgG and IgM were re-assessed by ELISA after IgG depletion.

### Comprehensive Metabolic Panel analysis

The kinetics of fourteen serum analytes were analyzed with a Piccolo^® ^blood chemistry analyzer (Abaxis, Inc., Union City, CA) and Comprehensive Metabolic Reagent Discs, as per manufacturer's recommendations.

### Cytokine profiles

Profiles of eleven serum cytokines were analyzed with an Accuri C6^® ^benchtop cytometer (Accuri Cytometers Inc., Ann Harbor, MI) and an eBioscience FlowCytomix Human Th1/Th2 11-plex Kit (Bender MedSystems GmbH, Vienna, Austria). Additionally, VEGF-A, CRP, RANTES, IFN-α, and CD40L simplex kits were multiplexed for analysis of additional ligands. Serum aliquots collected and frozen throughout the experimental timeline were analyzed concurrently at the end of the study.

### Laboratory confirmation of LF

All serum samples were tested for LASV-specific Ag and IgM and IgG antibodies by ELISA, as outlined above. ReLASV NP was serially diluted and used to generate a standard curve in Ag-capture ELISA and LFI formats. Sera from previously diagnosed LF patients were used as IgM and IgG Ab-capture ELISA calibrators. As discussed in detail herein, patients who presented to the KGH LFW with a febrile illness were assessed by the following criteria: Patients were considered to have acute LF if a reaction above background levels developed on reLASV LFI modules and/or LASV Ag-capture ELISA, and/or LASV-specific RT-PCR generated a GPC gene-specific amplicon using oligonucleotides previously described by Olschläger et al. [13]. Subjects testing only as IgM+ using reLASV IgM Ab-cature ELISA were not considered acute LF cases and often were not administered a course of ribavirin. The ultimate decision to administer a course of ribavirin to Ag-IgM+ patients was at the discretion of the attending KGH LFW physician. Subjects testing only as IgG+ using reLASV IgG were also not considered acute cases, but rather were considered to have been previously exposed to LASV and were deemed to be suffering from a non-Lassa febrile illness. Similarly, patients who were dual IgM+IgG+ and LASV Ag- at the time of testing were considered to be suffering from an unrelated febrile illness. For these patients additional testing and disease diagnosis was performed, as per the capabilities available at the KGH.

### Assignment to LF status groups

Patients were assigned to one of five groups on the basis of LASV Ag status, IgM and IgG profiles, and outcome. Group assignment was largely based on real-time data at the time of testing or admission to the KGH, and in a few cases the course of illness was followed into early convalescence [11,13]. The five groups were designated as: (1) Lassa fever, non-fatal, Ag+ (LF NF; N = 19); (2) Lassa fever, fatal, Ag+ (LF F; N = 25); (3) uninfected or afebrile controls (normal; N = 15); (4) Lassa fever, non-fatal, follow up patients sampled at least 8 weeks after discharge (LF FU; N = 18); and (5) LASV Ag-, IgM+, febrile patients (NL FI IgM+; N = 21). An additional group was analyzed that was comprised of individuals admitted to KGH with a non-Lassa febrile illness and a nonfatal outcome, irrespective of LASV-specific Ig status (NL FI NF; N = 37). The status of LASV Ag and IgM and IgG antibody profiles in populations from historically non-endemic LF areas of Sierra Leone were determined by ELISA as outlined above, but cytokines and metabolic markers were not analyzed for this subset of donors (Moyamba normals [MOY NHS]: N = 101; Bombali normals [BOM NHS]: N = 13).

### Statistical methods

#### Detection and measurement

ELISA data were plotted as mean ± SD, where each mean was based on two replicates, and error bars were used to represent the standard deviations. Limits of detection and quantitation of LASV-specific IgM were based on the upper 95^th ^percentile obtained with a panel of sera from U.S. (USN) and Sierra Leonean donors without detectable LASV Ag, IgM, and IgG titers. Cytokine levels were calculated by applying curve fitting techniques to data generated with quantified standards for each analyte.

#### Statistical analysis

Box plots of IgM and IgG serum responses were generated for seven comparison groups: USN, MOY NHS, BOM NHS, LF FU, LF NF, LF F, and NL FI IgM+. Box plots of serum responses for IgM and IgG profiles were generated using the Vertex42 Microsoft Excel template [14]. The Kruskal-Wallis test was used to compare median serum responses among the six comparison groups, and subsequent pairwise comparisons were conducted using Dunn's post test. Wilcoxon's signed rank test was used to assess changes in median IgM and IgG serum levels between baseline and follow-up end points. Simple linear regression was used to assess the relationship between IgM and IgG serum level responses and the number of days post discharge. The response data for these regression models was log-transformed to conform to linear regression assumptions. The Kruskal Wallis procedure was used to compare median NP Ag serum levels among the LF FU, LF NF, and the LF F groups. Logistic regression models were used to make patient subgroup comparisons with respect to survival outcome. All of the statistical analyses were conducted using GraphPad InStat 3 (GraphPad Software, Inc., San Diego, CA) and the SAS System (SAS Institute, Inc., Cary, NC) [15]. The statistical significance threshold for all of the analyses was set at *p *< 0.05.

## Results

### Odds of fatal outcome by test result in suspected LF patients

Survival data from a database comprised of 1,909 suspected LF patients who were tested for LASV Ag and IgM at the KGH LFL from 2007 - 2011 is summarized in Table 1 (N = 546). The patients for whom we had survival outcome were grouped according to Ag and IgM status. Odds ratios revealed that febrile patients who presented to the KGH LFW with LASV-specific Ag were statistically more likely to die than febrile patients who had no detectable Ag. These data are irrespective of IgM status. Patients who were both Ag and IgM positive were 4.33 times more likely to die than those who presented with an IgM+ titer, but no LASV Ag (*p *< 0.01). Similarly, Ag+ patients with no IgM upon admission were 5.36 times more likely to die than those who presented with neither Ag nor IgM titers (*p *< 0.01). We found no statistical difference between patients who had the same Ag status, again, irrespective of IgM. Both Ag+ groups (Ag+IgM+, Ag+IgM-) were nearly equally likely to die (*p *= 0.83), while both Ag- groups (Ag-IgM+, Ag-IgM-) where similarly likely to live (*p *= 0.61). IgM status, therefore, does not appear to impact survival outcome. Only 5 patients of this large cohort have presented to the KGH LFW with an Ag+ IgM- IgG+ profile: Four patients survived and one succumbed.

**Table 1:** Odds of fatal outcome by LASV-specific NP Ag- and Ab-capture ELISA

Test Result	N (% fatal)	Comparison Group	**Adjusted OR (95% CI)**^**a**^	*p*
Ag+IgM+	35 (54)	Ag-IgM+	4.33* (2.01, 9.30)	< 0.01
Ag+IgM-	96 (56)	Ag-IgM-	5.36* (3.19, 9.00)	< 0.01
		Ag+IgM+	1.09 (0.50, 2.40)	0.83
Ag-IgM+	171 (22)	Ag-IgM-	1.13 (0.70, 1.83)	0.61
Ag-IgM-	244 (20)	-	-	-

^a ^Odds of fatal outcome relative to the comparison group; all Ors are adjusted for gender.

* Statistically significant at the 5% significance level.

Individuals who presented to the KGH LFW were tested for LF by Ag- and Ab-capture ELISA. We subsequently categorized individuals based on LASV Ag and Ab status. Fatality comparisons of Ag+IgM+, Ag+IgM-, Ag-IgM+, and Ag-IgM- individuals revealed that there was no significant difference between Ag-status groups irrespective of IgM status. Conversely, Ag+ patients were about 5 times more likely to succumb than Ag- patients irrespective of IgM status (*p *< 0.01).

### Ag levels differ significantly between fatal and non-fatal LF

**Figure 1:**
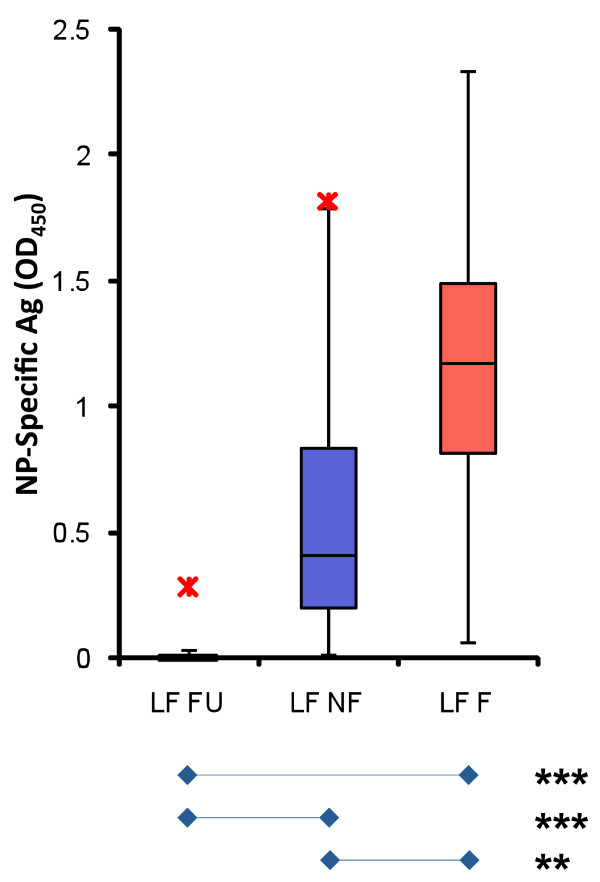
**Lassa virus antigen levels in LF patients**. Serum levels of LASV NP antigen in LF patients were quantitated by ELISA, using a sensitive caprine polyclonal antibody capture and detection sandwich method [11,12]. Follow-up convalescent patients primarily displayed undetectable levels of LASV antigen. Viral antigen levels between nonfatal (LF NF) and fatal LF cases (LF F) were significantly different at the time of admission or testing (N = 87, *p *< 0.01). The average time from onset of symptoms to admission was 8.0 ± 3.7 days for LF NF patients, and 10.4 ± 4.5 days for LF F patients. The difference between these times was not statistically significant (*p *= 0.07).

LASV NP Ag levels were measured in the serum of suspected LF patients by LFI, ELISA, PCR, or a combination of the three methods. Quantitative LASV NP Ag-capture ELISA was used to quantify levels of NP in LF patients. At the time of admission NP Ag levels between eventual fatal and non-fatal LF patients were statistically different (*p *< 0.01; Figure 1). Thus, Ag levels at time of admission could be used as a prognostic marker.

### Characteristics of LF patients, normal participants, and other non-LF febrile subjects

Characteristics of our study subjects are shown in Table 2 and Additional File 1. The average age for nonfatal and fatal LF patients was 28.3 and 18.7 years, respectively. No significant characteristic differences in characteristics between the LF F and LF NF groups were observed, except for duration of illness. Patients who survived to at least day three after admission at KGH LFW were at least 5.2 times more likely to survive (Table 2, Additional File 1). The odds ratio for the bleeding characteristic indicates a potential affect on survival outcome (OR = 5.1), but this study lacked a sufficient number of patients presenting bleeding symptoms to show statistical significance (Table 2, Additional File 1). A subset of patients presenting with either fever, bleeding, conjunctivitis, or a combination of symptoms, who tested negative for LASV Ag and positive for IgM (and mostly negative for LASV-specific IgG) were compared with Ag+ confirmed LF cohorts. The NL FI group was comprised of 37 febrile patients, with 21/37 (57%) registering significant LASV IgM titers (Additional File 1). We saw significant differences in the duration of illness and bleeding categories between LF F cases and NL FI IgM+ cases. The odds ratio indicated that an Ag-IgM+ (NL FI IgM+) individual who survived to at least day three of admission at the KGH was at least 2.48 times more likely to survive than an Ag+ individual (Table 2, Additional File 1). Also, an Ag+ patient who succumbed to LF was at least 1.09 times more likely to have bleeding symptoms than one who is negative for Ag (NL FI) and positive for IgM (Table 2, Additional File 1). A complete list of OR for each characteristic is displayed in Additional File 1.

**Table 2:** Characteristics of study subjects analyzed for cytokines and clinical chemistry^a^

Characteristic		LF F (N = 25)	LF NF (N = 19)	NL FI IgM+ (N = 21)
**Age**	< 15 yrs	11 (44)	5 (26)	6 (30) †
	15 - 40 yrs	14 (56)	10 (53)	10 (50)†
	> 40 yrs	0 (0)	4 (21)	4 (20)†
**Gender**	Male	12 (48)	7 (37)	6 (29)
	Female	13 (52)	12 (63)	15 (71)
**Duration of illness**	< 3 days	18 (72)	1 (5)^b^*	2 (11)†^d^*
	≥ 3 days	7 (28)	18 (95)	16 (89)†
**Major Signs**	Fever	24 (100) †	19 (100)	17 (94)†
	Bleeding	9 (36)	2 (11)^c^	1 (6)†^e^*
	Head swelling	7 (28)	4 (21)	1 (6)†
	Conjunctivitis	5 (20)	5 (26)	1 (6)†

a All results expressed as frequency (%), unless noted otherwise. Odds ratios (ORs) and their associated 95% confidence intervals were calculated using ordinary logistic regression.

b Odds of fatal outcome in LF F versus LF NF for the interval between date of admission and date of death or discharge is 46.3 (5.2, 415.6), which is significant.

c Odds of fatal outcome in LF F versus LF NF for the presence versus absence of bleeding symptoms is 5.1 (0.9, 27.4), which is not significant.

d Odds of fatal outcome in LF F versus NL FI IgM+ for the interval between date of admission and date of death or discharge is 14.4 (2.48, 80.68), which is significant.

e Odds of fatal outcome in LF F versus NL FI IgM+ for the presence versus absence of bleeding symptoms is 9.56 (1.09, 84.24), which is significant.

† Data unavailable for all observations.

* Significant at the 5% significance level.

The characteristics of three groups were compared based on antigen status and death. Ag+ LF patients were separated into two groups based on survival status. Age, gender and all but one of the major signs of LF appeared not to impact survival rates in LASV Ag+ patients. Odds ratio for duration of illness, however, was significantly different at the 5% significance level between those who survived LF and those who succumbed. Additionally, each LF group was compared to individuals who were suffering from a febrile illness that could not be definitively diagnosed as LF based on an Ag-IgM+ profile. There were no significant differences between LF NF and NL FI IgM+ groups amongst the various characteristics examined. Significant differences arose between LF F and NL FI IgM+ groups for duration of illness and bleeding. Ultimately, for both comparison groups, if individuals survived past day three after admission and initiation of treatment then their chances of survival increased significantly (OR = at least 5.2, statistically significant between LF F vs. LF NF; OR = at least 2.48, statistically significant between LF F vs. NL FI IgM+). Additionally, Ag+ patients who presented with bleeding symptoms were more likely to succumb (OR not statistically significant between LF F vs. LF NF; OR = at least 1.09, statistically significant between LF F vs. NL FI IgM+).

### Immunoglobulins in convalescent, nonfatal, and fatal LF

**Figure 2:**
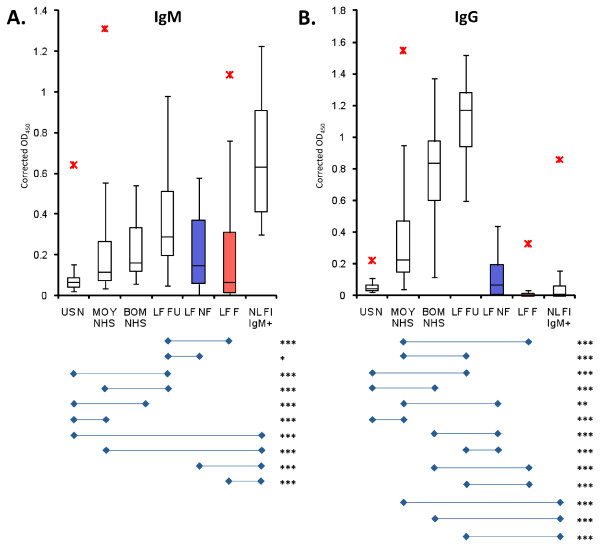
**IgM and IgG responses for normal donors, LF and NL febrile subjects**. Box plots of LASV-specific IgM (A) and IgG (B) levels determined by ELISA, are displayed as mean OD_450 _with corrected cutoff values based on the 95^th ^percentile of established negative control sera. Each display shows the minimum non-outlying value, three quartiles, maximum non-outlying value, and outlying values. The comparison groups include U.S. normals (US N), Moyamba district normals (MOY NHS), Bombali district normals (BOM NHS), convalescent LF follow-up patients (between 8 and 108 weeks post discharge [LF FU]), nonfatal acute LF cases (LF NF), fatal LF cases (LF F), and non-Lassa febrile illness with LASV-specific IgM only (NL FI IgM+). IgM and IgG levels for patients in the LF FU sera group were significantly higher than those in for all of the other comparison groups, save for those in the BOM NHS and NL FI IgM+ cohorts. There were no significant differences between LF NF and LF F cases for both IgM and IgG responses. Bombali sera showed relatively high levels of LASV-specific IgM and IgG, but these levels did not significantly differ from those for LF FU patients, despite their undiagnosed recent LF or other febrile illnesses. Outliers are indicated with red asterisks (*). Significant *p *values for pairwise comparisons are displayed as * *p *< 0.05; ** *p *< 0.01; and *** *p *< 0.001.

**Figure 3:**
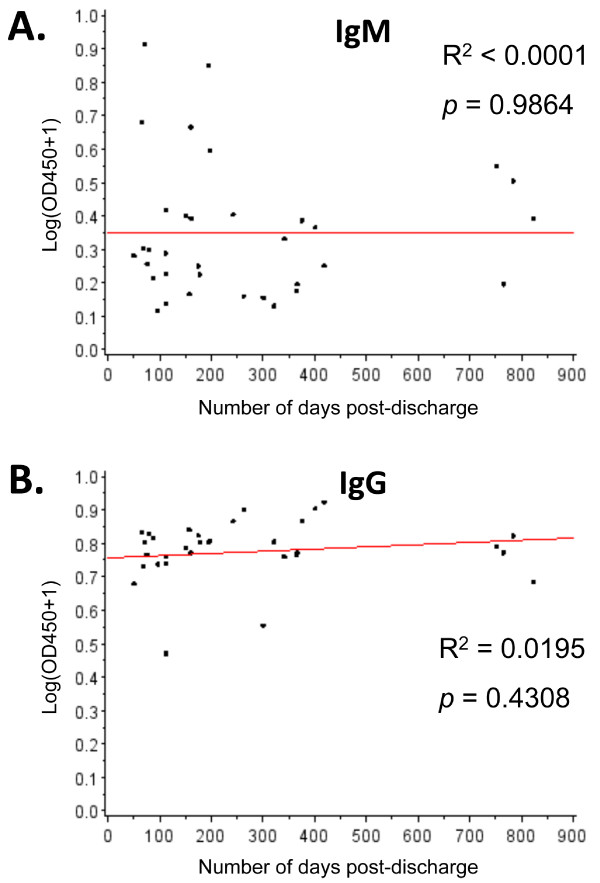
**Regression analysis for IgM and IgG responses against number of days post-discharge for convalescent LF patients**. Corrected mean OD_450 _values for LASV-specific IgM (A.) and IgG (B.) levels in LF convalescent patients did not reveal any dependence with time post-discharge for immunoglobulins responses. Hypothesis tests for the slope of each regression line revealed zero slopes for both profiles, suggesting that IgM and IgG responses for convalescent patients remained relatively constant after discharge. The fitted intercept for the regression line shown in (A.) was 0.46 (SE = 0.09), which showed prolonged elevation in IgM responses for convalescent LF patients. The fitted intercept of 1.14 (SE = 0.06) for (B.) was indicative of a prolonged mature humoral response (IgG) in convalescent LF patients.

The levels of LASV-specific serum IgM and IgG were compared among U.S. and West African normal controls (USN, MOY NHS and BOM NHS, respectively), surviving LF patients (LF FU), acute fatal and nonfatal LF patients, and febrile LASV Ag-IgM+ subjects (Figure 2). Additionally, LF FU IgM and IgG titers were measured for months to years into convalescence (Figure 3). These comparisons were aimed at establishing (1) how long into convalescence LASV-specific IgM levels persist; (2) if humoral responses between fatal and nonfatal LF differed significantly at time of admission and testing, thus serving as a prognostic marker; (3) if surviving LF patients mounted a significant humoral response during treatment that subsides upon recovery from acute infection. Studies on LF carried out between 2006 and 2011 revealed LASV-specific IgM levels persist in convalescence lasting from months to years after initial infection (Figure 3A). In this study, we analyzed the immunological and metabolic response in 34 LF convalescent patients who donated whole blood for analysis between 8 weeks and 2.2 years post-discharge. The mean time between discharge and follow-up analysis for these donors was 263 ± 221 days (ranging from 51 to 785 days). Corrected levels of LASV-specific IgM did not correlate linearly with time post discharge (R^2 ^< 0.0001, *p *= 0.986), suggesting that IgM does not subside rapidly following the rise of IgG titers in convalescence (Figure 3B). Similarly, LASV-specific IgG levels did not correlate linearly with time post discharge (R^2 ^= 0.0195, *p *= 0.431). A moderate to high level of IgG was recorded in all follow-up sera (Figure 2B, 3B).

### LASV-specific Ag, IgM and IgG levels in normal donors

Kenema district is a highly endemic LF region of Sierra Leone; therefore, collecting a representative sample population of Sierra Leonean normal controls in Kenema with low exposure to LASV was not possible. Instead, a panel of 101 sera samples was collected in April 2010 from individuals in the Southwestern province of Moyamba, described in the literature as a non-endemic region for LF [2,8,16-23], for analysis of LASV Ag, IgM, and IgG profiles (Additional File 2A,B, Figure 2). Additionally, 13 sera samples were obtained from donors in the Northeastern province of Bombali, which is also purported to be a non-endemic region for LF in Sierra Leone (Additional File 2A,B, Figure 2) [2,8,16-23]. All donors were interviewed prior to blood drawings and all reported normal health status with no recent febrile illnesses. All 101 sera from Moyamba and 13 sera from Bombali were negative for LASV NP Ag (Additional File 2C). Conversely, after applying a correction factor based on the limit of detection (LoD) of the assays, 28 (27.7%) of the Moyamba samples tested positive for IgM, 63 (62.4%) tested positive for IgG, and 18 (17.8%) tested positive for both IgM and IgG (Additional File 2C). For the 13 Bombali samples, 3 (23.1%) tested positive for IgM, 12 (92.3%) tested positive for IgG, and 3 (23.1%) tested positive for both IgG and IgM (Additional File 2C). Specificity of the IgM and IgG ELISA binding data was confirmed by western blot analysis on recombinant LASV antigens with normal Moyamba and Bombali sera and by homologous antigen competition ELISA (Garry et al, unpublished data). Additionally, depletion of IgG from serum abrogated detection of LASV-specific IgG binding to antigens by ELISA (NP, GPC, Z protein combinations), but did not significantly affect relative levels if IgM binding to the same proteins (data not shown). Moreover, a blinded panel of sera from 50 female and 50 male donors obtained from U.S. sources tested negative for LASV NP Ag and virus-specific IgM and IgG Ab (Figure 2).

### Pre-existing humoral response to LASV in normal populations compared to diagnosed LF patients

IgM levels were not significantly different between LF FU and Bombali donors, but they were significantly higher for LF FU donors than for Moyamba donors (*p *< 0.001; Figure 2A). Similarly, LF FU patients had higher IgG levels than Moyamba normal donors (*p *< 0.001) but they did not differ from Bombali donors (Figure 2B). Furthermore, LF FU patients showed significantly higher IgG levels than LF NF (*p *< 0.001) and LF F (*p *< 0.001) patients at the time of diagnosis (Figure 2B). These data suggest that (1) most symptomatic and acute LF patients presenting to KGH LFW are naïve to LASV; (2) exposure to LASV in Sierra Leone is more prevalent than previously reported and remains mostly undiagnosed; (3) regions of Sierra Leone previously considered non-endemic for LF revealed a significant level of LASV-specific IgM and IgG prevalence.

### Circulating inflammatory mediators and metabolic panel in Lassa fever

**Figure 4:**
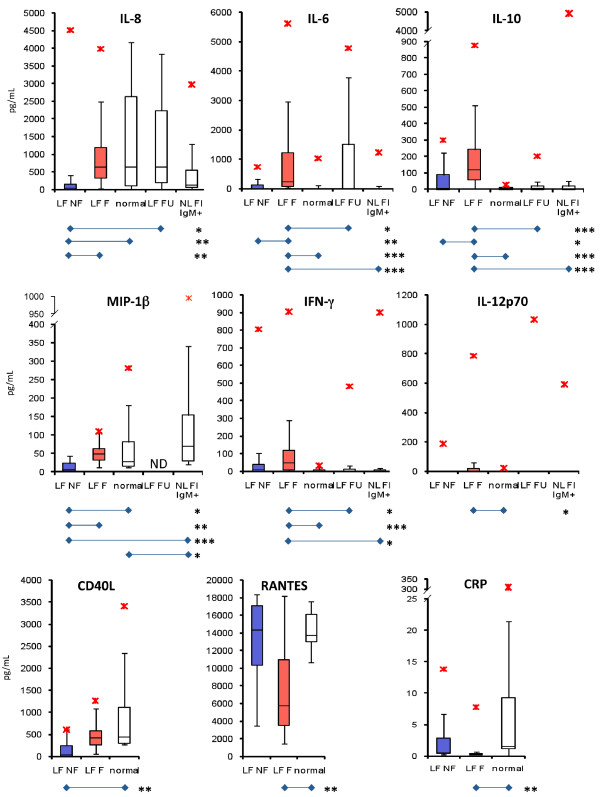
**Levels of relevant cytokines in LF pathogenesis**. Serum levels of IL-8, Il-6, IL-10, and MIP-1β showed significant differences between LF NF and LF F patients on admission. For LF NF patients, IL-8 levels were significantly reduced when compared to LF F, normal, and LF FU subjects. Elevated levels of IL-6 and IL-10 were recorded in LF F patients, but were significantly lower in LF NF subjects. MIP-1β was significant reduced in LF NF only when compared to LF F patients. Interferon-γ was significantly higher for fatal LF patients than normal donors and follow-up controls, but IFN-γ levels did not differ between fatal and nonfatal LF patients. Similarly, IL12p70 levels were significantly elevated in LF F when compared to normal donors, but did not differ from the other comparison groups. CD40L was significantly reduced in LF NF when compared with normal controls, but not in LF F. Cytokine levels were relatively similar between LF NF and NL FI IgM+ groups, with the exception of MIP-1β, which was elevated in the later cohort. Outliers are denoted with red asterisks (*). Significant *p *values for pairwise comparisons are denoted as * *p *< 0.05; ** *p *< 0.01; and *** *p *< 0.001.

**Figure 5:**
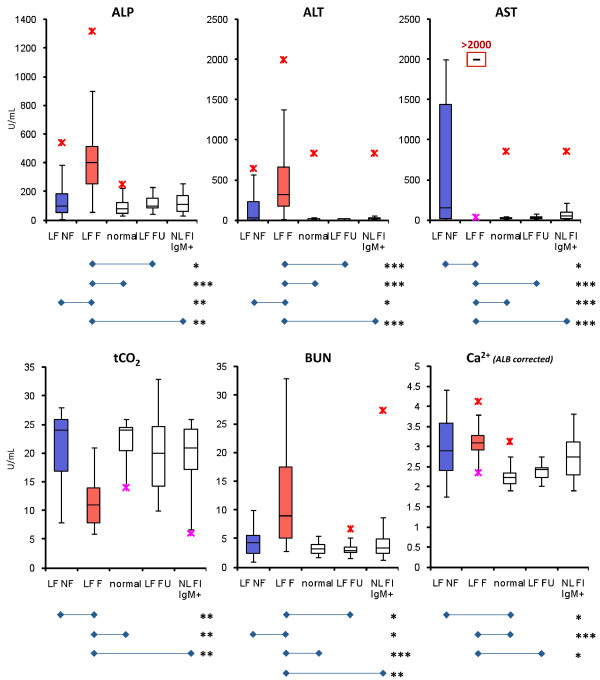
**Relevant metabolic indicators in LF pathogenesis**. Hepatic enzymes ALP, ALT, and AST were highly elevated in LF patients, particularly for LF F patients. AST levels were the best prognosticator in LF, with nearly all fatal cases showing extremely high levels. Dissolved tCO_2 _levels were significantly lower than normal for LF F patients, whereas BUN levels were significantly higher than normal. Serum calcium levels (corrected for serum albumin levels) were elevated in LF patients regardless of eventual outcome, but no differences in elevation were observed between acute LF groups. Lower and upper outliers are denoted with pink and red asterisks (*), respectively. Significant *p *values are denoted as * *p *< 0.05; ** *p *< 0.01; *** *p *< 0.001.

Evaluation of pro- and anti-inflammatory cytokines, chemokines, growth factors, and disease state metabolite indicators revealed statistically significant to highly significant differences between LF patients and non-LF controls in the levels of interleukin (IL) -10, IL-8, IL-6, macrophage inflammatory protein 1 beta (MIP-1β) (Figure 4), blood urea nitrogen (BUN), total carbon dioxide (tCO_2_), calcium (Ca^2+^) corrected for albumin (ALB), alkaline phosphatase (ALP), alanine aminotransferase (ALT), and aspartate aminotransferase (AST) (Figure 5) in LF patients compared to non-LF controls. Other markers, such as interferon gamma (IFN-γ) (Figure 4), IL-4, and creatinine (CRE) (data not shown) showed wider ranges of increased expression in fatal cases than nonfatal cases, but the differences in median expression levels were not statistically different. Similarly, statistical differences were not observed between fatal and nonfatal LF cases for levels of IL-1β, which tended to be lower than normal for both fatal and nonfatal LF groups (data not shown). IL -6 and -10 were significantly higher for fatal LF cases than normal, nonfatal, and follow-up donors, but differences were not observed between nonfatal LF and normal controls (Figure 4). MIP-1β was significantly less for only fatal cases as opposed to nonfatal cases, but did not differ between fatal cases and normals (Figure 4). Cytokines IL-12p70, IL-2, IL-5, TNF-α, TNF-β, and IFN-α did not appear to be influential in the pathogenesis of LF (Figure 4 and data not shown). Albumin (ALB) levels were not significantly different between fatal and non-fatal LF, but in both cases were significantly lower than in normal controls (data not shown). Total serum protein (TP) levels, however, were significantly less than normal for fatal LF cases (data not shown). Total bilirubin (TBIL), and levels of chloride (Cl-), potassium (K+), and sodium (Na+) ions were not significantly dysregulated in LF (data not shown). BUN, ALP, AST, and ALT were all highly elevated in fatal LF, but not significantly different between non-fatal LF and normal subjects (Figure 5). Calcium (Ca^2+^) levels in non-fatal and fatal LF were significantly higher than normal after correcting for albumin levels (Figure 5). The most significant prognostic marker of fatal outcome in LF was highly elevated AST. All LF subjects who met with a fatal outcome in this study with the exception of one low outlier presented with extremely high levels of AST (Figure 5). The LF convalescent group displayed cytokines and metabolic markers similar to those recorded in normal individuals, with the exception of IL-6, which showed a higher range than normal donors, but the median IL-6 level for the convalescent group was not statistically significant (Figure 4).

### LASV IgM+ Ag- febrile subjects present with inflammatory and metabolic profiles different from those of LF F but not LF NF patients

NL FI IgM+ subjects presented to the hospital with fever, and in some instances bleeding and conjunctivitis, all of which are major symptoms of LF (Table 2). These patients registered low LASV-specific IgG titers (Figure 2B), and all were negative for viral antigen. Cytokine profiles were unremarkable when compared to LF NF, with the exception of MIP-1β, which was significantly elevated in NL FI IgM+ subjects (Figure 4). No significant differences were observed in levels of metabolic indicators between LF NF, NL FI IgM+, and normal donors (Figure 4, 5). Conversely, the LF F and NL FI IgM+ groups differed significantly in most cytokines and metabolic indicators, with the exception IL-6, MIP-1β, IL12p70, and corrected Ca^2+ ^levels (Figure 4, 5). The most remarkable trait recorded in the NL FI IgM+ patient subset was the absence of detectable LASV Ag or nucleic acid. The attending physician closely monitored the 21 patients in this group after testing for LASV Ag and immunoglobulins, but only 4 were treated with ribavirin. Notably, treatment of patients is at the discretion of the attending physician; febrile and LASV Ag- IgM+ patients presenting with additional symptoms of LF infection may be treated with ribavirin. In this group 3 subjects expired shortly after admission (N = 2, 4 days to expiry; N = 1, 0 days to expiry), 4 were discharged (N = 2, 10 days; N = 1, 4 days; N = 1, 3 days), and 14 were not admitted.

## Discussion

Historically, LASV viremia has been the primary marker of acute infection as assessed by Ag-capture ELISA, LASV RNA detected by RT-PCR or virus culture (the latter serving as the "gold standard"). Alternatively, elevated LASV-specific IgM has served as a surrogate marker of a recent infection when LASV Ag could no longer be detected [24]. With little known about LASV-specific humoral immune responses and immunopathology, the rationale for considering IgM positivity as acute LASV infection was based on observations in other pathogenic infections in which a drop in antigen levels coincided with increasing IgM titers that class switched to a predominantly IgG status within weeks of initial infection. Given the low rate of IgM and IgG seropositivity in healthy blood donors from the United States, IgM seropositivity in non-Africans recently traveled to LF endemic regions of West Africa is a likely indication of a recent LASV infection. Results presented herein, however, suggest that LASV-specific IgM seropositivity in the absence of viremia should not be considered as a diagnostic marker for acute LF in the regions of West Africa where evidence of a high prevalence of LASV-specific seropositive status exists.

Because Kenema is historically endemic for LASV, we looked to the Northeastern and Southwestern districts of Bombali and Moyamba, respectively, to collect a panel of normal Sierra Leonean serum. These districts lie within reportedly non-endemic areas so exposure to LASV should be low. The donors from these two regions reported normal health at the time of blood draw without febrile episodes in the recent past, and had not travelled to the Eastern provinces where LF is endemic. Contrary to our expectations, a significant percentage in each population had LASV-specific IgM and IgG titers indicating a past infection with LF. Our findings challenged past reports claiming that LF is highly endemic primarily in the Eastern districts of Sierra Leone, and is largely absent in the country's Northern and Southern regions (Additional File 2A, B). It is noteworthy that in the past one year a significant number of suspected LASV cases from Bombali have been referred to the KGH, with several being confirmed as LF [12]. Identification of suspected LF cases has been made possible by increased awareness of the disease by medical staff in Bombali district hospitals and the availability of rapid diagnostics provided by the VHF consortium.

These surprising results prompted us to reevaluate the categorization and analysis of Ag and IgM status in a suspected LF patient database compiled at the KGH LFL between 2006 and 2011. To determine whether IgM status could be used as a diagnostic marker, we compared the odds ratios of a group of LASV Ag+ patients and LASV Ag- patients. This revealed that, regardless of IgM status, the fatality rate was significantly higher in Ag+ patients compared to Ag- patients (Ag+IgM+ v. Ag-IgM+ OR = 4.33, *p *< 0.01; Ag+IgM- v. Ag-IgM- OR = 5.36, *p *< 0.01). Additionally, we found that positive LASV IgM seropositivity is not a reliable marker of acute LF base on the adjusted odds ratio of 1.13 in Ag-IgM+ (N = 171) versus Ag-IgM- (N = 244) in suspected LF patients (*p *= 0.61, Table 1). Conversely, Ag+ status, irrespective of IgM status, was indicative of a poor outcome, with approximately 55% of patients succumbing to LF. This percentage is higher than the historical and consistently reported death rate of approximately 15-20% because we eliminated a large population of patients that could not be considered as acute infections with LASV [25-28]. We believe that 55% is a more accurate fatality rate based on the prolonged LASV-specific IgM response found in convalescent patients months to years following LASV infection (Figure 2) [11,12]. Our data show that detectable LASV viremia assessed by Ag seropositivity is highly correlated with acute viral infection, while IgM shows no such correlation.

There are several potential interpretations for sustained LASV-specific IgM titers [29-33]: 1. IgM may be indicative of a recent infection for which class switching has not fully occurred; 2. A prolonged and as of yet largely uncharacterized inhibition of class switching could be prevalent in LASV infections (Grove, Garry et al., unpublished data); 3. Sustained IgM titers could be generated by IgM+ memory B cells; 4. Impaired CD4+ T helper lymphocyte function during LASV infection, with long-term impact on class switching may be central to humoral and cellular immunity aspects in LF; (5) Individuals may be infected with LASV but clear viremia without treatment, and seek medical intervention too late for detection of virus antigen by LFI, ELISA, and nucleic acid by PCR.

Evidence for these mechanisms can be found in the literature. Non-neutralizing virus-specific IgM were recently analyzed and deemed crucial for impedance of viral persistence in LCMV infection (the prototypic Old World arenavirus), suggesting that IgM-producing cells have remained largely obscure and underappreciated in the characterization of arenaviral infections [34]. Additionally, the late appearance of neutralizing antibodies in arenaviral infections has been tied to high viral antigen-to-B cell ratios and low T cell help, which resulted in a normal IgM response but reduced the efficiency of class switching [35]. Lowering the antigen-to-B cell ratio and increasing T cell help resulted in rescuing of class switching and emergence of neutralizing IgG specificities. Thus it is possible that a normal early IgM response in LASV infections is followed by an impaired T helper cell response with sustained IgM production and eventual maturity of the producing B cell subset into an IgM+ B cell memory population. The role of IgM and T-helper lymphocytes have been studied in controlled cynomolgus macaque models of SIV infection [36]. These studies noted a strong inverse correlation of the immunoglobulin and CD4+ T helper cell counts after the primary peak of IgM response, reflecting the prevalence of mature plasma cells that have not undergone class switching. Moreover, a strong correlation was observed in the same studies between pre-infection immune status and disease progression. It is therefore possible in arenaviral infections, as in the SIV model, that a normal, pre-infection CD4+ T helper cell threshold may regulate normal B cell responses, with concomitant class switching, emergence of strong neutralizing IgG titers, and quiescence or elimination of short lived IgM-producing plasma cells. We have recently reported on two cases of severe LF [11,12] that registered increasing IgM titers throughout hospitalization and for months into convalescence. Virus-specific IgMs were invariably paralleled by rising, albeit temporally delayed, IgG titers. We have observed similar circulating Ig patterns in numerous other LF cases, with rising IgG titers following an initial IgM response. Interestingly, in the 21 NL FI IgM+ subjects we did not record LASV-specific IgG titers, despite prolonged hospitalization and multiple sera testing for a subset of patients (Figure 2 A, B). Therefore, it appears that in this group of patients LASV-specific IgM responses were indicative of a previous infection with muted class switching.

In the Ag positive subset of patients, remarkable differences were noted in cytokine and metabolite levels that could be used as early prognosticators in LF. Our results have confirmed historical reports demonstrating significant hepatic and renal dysfunction in LF, namely the high levels of ALP, ALT, and AST [30-39]. However, not all of our cytokine and metabolite observations follow the historical observations from the literature. Mahanty et al. previously reported that elevated levels of IL-8 and IP-10 correlated with positive outcomes in LF [24], while we observed that low levels of IL-8 and other cytokines to be correlates of survival outcome. The frequent exposure of populations to parasitic infections is a likely factor in the elevated levels of recorded IL-8 in the sampled normal and non-Lassa febrile groups, a phenomenon not commonly observed in U.S. blood donors (Figure 4). Although there was not a significant reduction in the overall levels of IL-8 between normal donors and fatal LF patients, a significant drop in the levels of this cytokine was observed in all patients who survived LF (excepting a single, high outlier). Thus our data from this report and others [37-39] suggest that a regulated quiescence of the inflammatory response, in addition to a robust humoral immune response may be central to a successful outcome in symptomatic acute LF. In addition to IL-8, low levels of IL-6, IL-10, MIP-1β, and CD40L were predictive of survival. A subset of individuals presenting with a non-Lassa febrile illness that may have registered LASV-specific IgM titers were not included in our acute LF study group, but were included in the acute LF population studied by Mahanty et al. [24]. The differences between our studies and those by Mahanty et al. [24] and others may therefore rest on the parameters employed in classifying acute LF.

Our analysis of a nonfatal non-Lassa febrile illness (NF NL FI) study group revealed levels of IL-8 similar to those in the LF NF and LF F groups, despite observing increased levels of the inflammatory mediator in some subjects (Figure 4). The only inflammatory marker analyzed in our studies that differentiated LF from other FIs was IL-6, which was increased in LF F relative to all other comparison groups (Figure 4). Interestingly, in the NL FI IgM+ (and IgG- Ag-) group we observed reduced levels of IL-8 and elevated levels of MIP-1β, as well as metabolic indicators that did not significantly differ from those in LF NF. A comparable profile of inflammatory mediators was observed in a study of LF pathogenesis in cynomolgus macaques, which revealed that elevated levels of IL-6 conferred a poor prognosis, while IL-8 and IL-10 responses were largely absent [40].

Together, these data indicate that upon admission to the hospital with suspicion of LF, a profile of low to moderate IL-6, -8, -10, MIP-1β, CD40L, BUN, ALP, ALT, and AST levels predict a positive outcome following treatment with a full regimen of ribavirin, fluids management, antibiotics, and other appropriate medical intervention. Conversely, AST levels greater than 2000 U/mL are almost always indicative of a poor survival outcome. In addition to high AST, combined elevated IL-6, -8, -10, BUN, ALP, ALT, and reduced tCO_2_, Ca^2+^, RANTES, and CRP levels provide a statistical basis for poor prognosis. Two recent extensively-characterized reports of hemorrhagic LF in Sierra Leone with positive outcome presented to the KGH LFW with AST > 2000 U/mL and low levels of IL-6, -8, and in one case elevated IL-10 [11,12]. Based on the data presented herein, the prognosis for both patients would have been poor, yet, with a relatively short timeframe from onset of symptoms to presentation to the KGH LFW (6 and 7 days, respectively) and proper medical interventions, both patients survived. The data collected in these studies generated statistically relevant correlates of LF outcome, but have also exposed gaps in our current understanding of relevant biomarkers for LF. The observed pronounced quiescence of the inflammatory response in surviving LF patients may present new opportunities for future disease treatment and management. It is conceivable that administration of anti-inflammatory drugs to quiesce the cytokine storm observed in LF may complement the beneficial effects of reducing viral loads with ribavirin treatment, thus increasing survival rates in acutely infected patients.

Limitations of this study include lack of randomization, limited number of fatal Lassa cases, potential patient by treatment interaction, and the limited duration of the study. Random assignment of subjects to comparison groups was not possible due to the uncontrollable nature of the outcome, and random selection of patients was impractical due to the limited number of fatal Lassa cases. As a result, some of the results that we observed may be due to inherent characteristics of our study subjects. This is perhaps most evident in the 13 normal donors from Bombali that registered greater than 90% IgG reactivity to LASV antigens. It is important to note that these limited studies were not designed to ascertain levels of IgM and IgG seroprevalence in Sierra Leonean populations. Instead, through the continuous analysis of incoming sera from patients and random collection of normal samples in two districts outside historically endemic regions, it was established that LASV infections in humans might be more prevalent across Sierra Leone than previously reported. In the case of Bombali donors we may have collected sera from a cluster of individuals who may have had an unknown exposure bias to LASV, thus registering high levels of IgG (and possibly IgM) to viral antigens. We suspect our study subjects are more likely to have multiple exposures to LASV than subjects from previous studies due to the increasing prevalence of the disease. Due to the small sample sizes for each of the comparison groups, we were unable to adjust for confounding variables, which makes the generalization of these findings inappropriate, particularly for those outside of Sierra Leone. Although all of the patients in this study were subjected to the same treatment protocol, it is unknown whether the treatment was administered in a consistent manner and whether patients responded differently to the ribavirin treatment. A randomized trial on the effect of ribavirin would be beneficial to future studies on Lassa fever. It is worth noting that there is a temporal component for the outcomes investigated in this study that is not well understood.

Due to our characterization of a prolonged IgM response in convalescent LF patients, high prevalence of IgM seropositive in healthy normal controls, and the failure of most IgM only suspected cases to display a dysregulated metabolic and inflammatory cytokine profile similar to LASV-specific Ag+ patients regardless of IgM status, we suggest that the traditional paradigm for diagnosis of acute LF in West Africa should be reconsidered and changed. Rather than diagnosing an Ag+ and/or IgM+ result as acute LF, only in the subset of patients displaying LASV viremia, as determined by LFI and Ag-capture ELISA, with confirmation by RT-PCR should be definitively categorize a patient as being acutely infected with LASV. Patients that presented with symptoms indicative of potential LF and who do not test positive for viremia by any of the three methods employed while presenting with IgM and/or IgG titers should not be categorized as acute LF. Unfortunately, ultimate diagnosis of the non LF febrile illness for these patients will often not be possible due to low resources and lack of diagnostics for additional suspected infectious agents. Though, the new prognostic immune correlates of LF identified in this study will allow the scientific community to better understand, monitor, and possibly treat and prevent the disease.

## Competing interests

This work was performed as partial fulfilment of Ph.D. dissertation requirements for Jessica N Grove.

## Authors' contributions

Conceived and designed the experiments: LMB, JNG, MLB, RFG. Performed the experiments: LMB, JNG, MLB, AG, MF, MM. Analyzed the data and critically reviewed the manuscript: LMB, MLB, JNG, JGS, DSG, RFG. Performed critical statistical analysis of the data: JGS. Wrote the manuscript: LMB, JNG, JGS, RFG. All authors read and approved the manuscript.

## Supplementary Material

Additional file 1 **Complete characteristics of study subjects analyzed for cytokines and clinical chemistry**. An expanded set of groups was analyzed for age, gender, duration of illness, and major signs. Corresponding odds ratios are shown, and asterisks (*) indicate significance at the 5% level. Click here for file

Additional file 2 **Map of West Africa displaying calculated rates of LASV Ag, IgM, IgG, and dual antibody in sera samples obtained from Sierra Leonean Districts of Moyamba and Bombali**. (A) Districts of Sierra Leone: the historically hyperendemic districts of Kenema and Kallahun are circled in blue, and the Northern and Southern districts of Bombali and Moyamba are underlined in red. A map outlining Sierra Leone's four provinces is shown in (B). The relative locations in Sierra Leone where panels of normal sera study samples were collected are boxed in red. Antigen and immunoglobulin rates for locations sampled in this study are outlined in insets. Numbers of sera analyzed from each region are noted (N). Serological evidence of LF has been reported in Senegal and Mali (denoted with solid blue circles), and outbreaks are commonly reported in endemic regions of Sierra Leone, Guinea, and Liberia (denoted with solid red circles). The relative sub-Saharan geographical boundary for LF is outlined by the thick transparent orange line dissecting Guinea and Southern Mali [17]. Source of maps: A. and B. http://commons.wikimedia.org/wiki/Atlas_of_Sierra_Leone; C. Google maps. Click here for file
