# Supplementary material for: Emerging trends in Lassa fever: redefining the role of immunoglobulin M and inflammation in diagnosing acute infection
Source: Virol J. 2011 Oct 24;8:478. doi: 10.1186/1743-422X-8-478 (PMC3223505; doi:10.1186/1743-422X-8-478)
Supplement: Additional file 1 — Complete characteristics of study subjects analyzed for cytokines and clinical chemistry. An expanded set of groups was analyzed for age, gender, duration of illness, and major signs. Corresponding odds ratios are shown, and asterisks (*) indicate significance at the 5% level. [file 1743-422X-8-478-S1.DOC]

**Additional File 1. Complete characteristics of study subjects analyzed for cytokines and clinical chemistry^a^**

|  | **Lassa cases (Ag+)** | | |  | **Febrile Illness** | **Ag+ and Ag- comparisons**  **OR (95% CI)^c^** | |
| --- | --- | --- | --- | --- | --- | --- | --- |
| **Characteristic** | **LF F**  **(n = 25)** | **LF NF (n = 19)** | **OR (95% CI)^b^** |  | **NL FI**  **IgM+**  **(n = 21)** | **LF F vs.**  **NL FI IgM+** | **LF NF vs.**  **NL FI IgM+** |
| **Age** < 15 yrs | 11 (44) | 5 (26) | 1.6 (0.4, 6.0) |  | 6 (30) † | 1.31 (0.36, 4.73) | 1.0 (referent) |
| 15 – 40 yrs | 14 (56) | 10 (53) | 1.0 (referent) |  | 10 (50)† | 1.0 (referent) | 1.2 (0.27, 5.25) |
| > 40 yrs | 0 (0) | 4 (21) | NA |  | 4 (20)† | NA | 1.2 (0.19, 7.44) |
|  |  |  |  |  |  |  |  |
| **Gender** Male | 12 (48) | 7 (37) | 1.6 (0.5, 5.4) |  | 6/ (29) | 2.31 (0.68, 7.89) | 1.46 (0.39, 5.51) |
| Female | 13 (52) | 12 (63) | 1.0 (referent) |  | 15 (71) | 1.0 (referent) | 1.0 (referent) |
|  |  |  |  |  |  |  |  |
| **Duration** < 3 days | 18 (72) | 1 (5) | 46.3* (5.2, 415.6) |  | 2 (11)† | 14.14* (2.48, 80.68) | 1.0 (referent) |
| **of illness^d^** ≥ 3 days | 7 (28) | 18 (95) | 1.0 (referent) |  | 16 (89)† | 1.0 (referent) | 3.27 (0.27, 40.47) |
|  |  |  |  |  |  |  |  |
| **Major**  Fever | 24 (100) † | 19 (100) | NA |  | 17 (94)† | NA | NA |
| **Signs**  Bleeding | 9 (36) | 2 (11) | 5.1 (0.9, 27.4)^e^ |  | 1 (6)† | 9.56* (1.09, 84.24)^h^ | 2.00 (0.17, 24.19)^h^ |
| Head swelling | 7 (28) | 4 (21) | 1.5 (0.4, 6.3)^f^ |  | 1 (6)† | 6.61 (0.73, 59.24)^i^ | 4.53 (0.46, 45.16)^i^ |
| Conjunctivitis | 5 (20) | 5 (26) | 1.4 (0.3, 5.6)^g^ |  | 1 (6)† | 4.25 (0.45, 40.01)^j^ | 6.07 (0.63, 58.22)^j^ |

a All results expressed as frequency (%), unless noted otherwise. Odds ratios (ORs) and their associated 95% confidence intervals were calculated using ordinary logistic regression. NA indicates insufficient data for calculating odds ratio.

b Expressed as the odds of a fatal outcome for the LF F and LF NF groups

c Expressed as the odds of testing Ag+ for defined group comparisons

d Defined as the interval between date of admission and date of discharge or death.

e Odds of fatal outcome for the presence versus the absence of bleeding symptoms.

f Odds of fatal outcome for the presence versus the absence of head swelling symptoms.

g Odds of fatal outcome for the absence versus the presence of conjunctivitis symptoms.

h Odds of testing Ag+ for the presence versus the absence of bleeding symptoms.

i Odds of testing Ag+ for the presence versus the absence of head swelling symptoms.

j Odds of testing Ag+ for the absence versus the presence of conjunctivitis symptoms.

† Data unavailable for all observations.

* Significant at the 5% significance level.
