# Supplementary figures and images for: Emerging trends in Lassa fever: redefining the role of immunoglobulin M and inflammation in diagnosing acute infection
Source: Virol J. 2011 Oct 24;8:478. doi: 10.1186/1743-422X-8-478 (PMC3223505; doi:10.1186/1743-422X-8-478)

A.

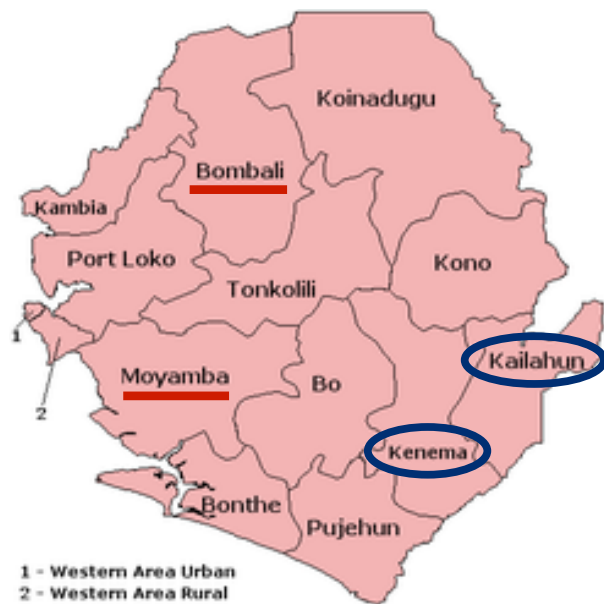

B.

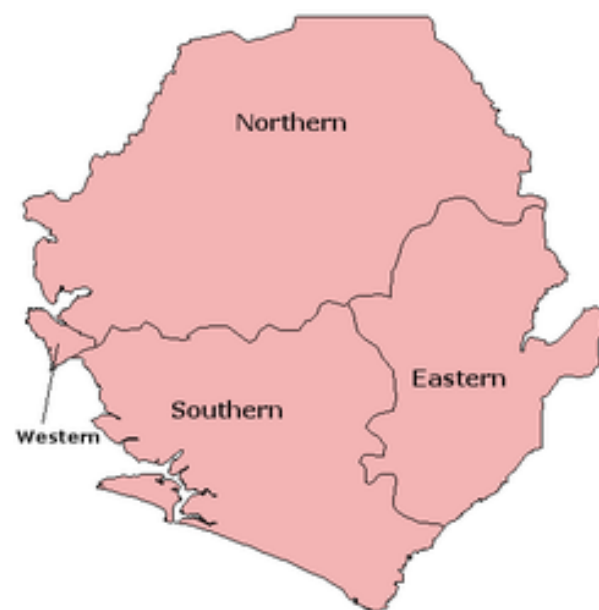

C.

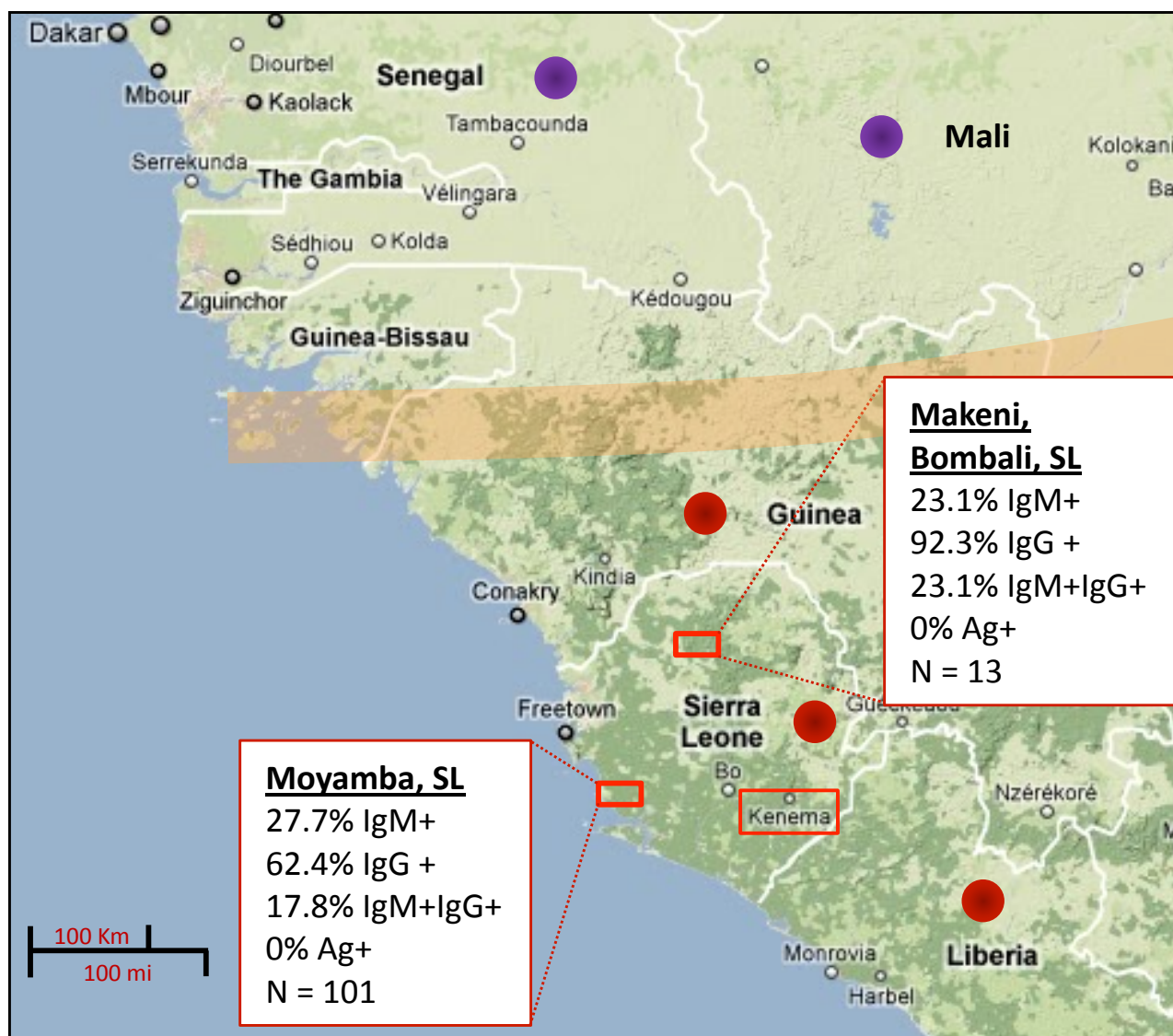

Supplement: Additional file 2 — Map of West Africa displaying calculated rates of LASV Ag, IgM, IgG, and dual antibody in sera samples obtained from Sierra Leonean Districts of Moyamba and Bombali. (A) Districts of Sierra Leone: the historically hyperendemic districts of Kenema and Kallahun are circled in blue, and the Northern and Southern districts of Bombali and Moyamba are underlined in red. A map outlining Sierra Leone's four provinces is shown in (B). The relative locations in Sierra Leone where panels of normal sera study samples were collected are boxed in red. Antigen and immunoglobulin rates for locations sampled in this study are outlined in insets. Numbers of sera analyzed from each region are noted (N). Serological evidence of LF has been reported in Senegal and Mali (denoted with solid blue circles), and outbreaks are commonly reported in endemic regions of Sierra Leone, Guinea, and Liberia (denoted with solid red circles). The relative sub-Saharan geographical boundary for LF is outlined by the thick transparent orange line dissecting Guinea and Southern Mali [17]. Source of maps: A. and B. http://commons.wikimedia.org/wiki/Atlas_of_Sierra_Leone; C. Google maps. [file 1743-422X-8-478-S2.PDF]
